# P25 - Hypersensitivity/adverse reactions to antituberculosis drugs – a case report

**DOI:** 10.1186/2045-7022-4-S1-P80

**Published:** 2014-02-28

**Authors:** Estefânia Barrosa Maia, Teresa Reis Silva, Nelson Neves, Miguel Félix, Carla Chaves Loureiro

**Affiliations:** 1 Hospital Pediátrico, Centro Hospitalar e Universitário de Coimbra, Coimbra, Portugal

## Background

Tuberculosis remains a common condition in children worldwide. Adequate treatment requires multiple antibiotics for several months. Hypersensitivity/adverse reactions to antituberculosis drugs can interfere with continuity of treatment and result in severe reactions.

## Case report

A ten years old boy was admitted for pleural tuberculosis (negative cultures, positive quantiferon assay, and father under treatment for tuberculosis). On the tenth day of treatment with isoniazid, rifampicin and pyrazinamide he develloped an exuberant urticarial rash, facial oedema, fever, myalgias, oliguria and, later, conjunctival hyperemia. Laboratory results included low platelet count, hypoalbuminemia and hyponatremia. Gradual improvement occured after suspension of treatment. One month later, drugs were gradualy re-introduced (1 drug/week) but several reactions occurred: anaphylaxis 5 hours after rifampicin administration and generalized macular rash after pyrazinamide. Both drugs were suspended. Isoniazid caused an initial light generalized macular exanthema. Streptomycin, ethambutol and ciprofloxacin were introduced and well tolerated. In subsequent evaluation the child was asymptomatic, with normal analytic results and radiologic improvement. After 6 months of successful treatment, he was tested in detail for reactions to the implicated drugs.

## Discussion

Reactions to antituberculosis drugs occur with variable frequency and severity, and may result from a single drug or the interaction of two or more. In this case, a severe reaction to the association of rifampicin and isoniazid seems to have occurred. Haematological disorders associated with rifampicin are a contraindication to reintroduction/tolerance induction of the drug so skin tests were not performed. Skin prick tests and intradermal tests to isoniazid were negative. Pyrazinamide reaction was only cutaneous and there are no validated tests to confirm diagnosis. This case shows the difficulty in continuity of treatment when hypersensitivity/adverse reactions occur.

